# Stent grafts improved patency of ruptured hemodialysis vascular accesses

**DOI:** 10.1038/s41598-021-03933-1

**Published:** 2022-01-07

**Authors:** Min-Tsun Liao, Chien-Ming Luo, Ming-Chien Hsieh, Mu-Yang Hsieh, Chih-Ching Lin, Wei-Chu Chie, Ten-Fang Yang, Chih-Cheng Wu

**Affiliations:** 1 Division of Cardiology, Department of Medicine, National Taiwan University Hospital Hsinchu Branch, Hsinchu, Taiwan, ROC; 2 College of Medicine, National Taiwan University, Taipei, Taiwan, ROC; 3 Division of Cardiovascular Surgery, Department of Surgery, National Taiwan University Hospital, Hsinchu Branch, Hsinchu, Taiwan, ROC; 4 Institute of Biological Science and Technology, National Yang Ming Chiao Tung University, Taipei, Taiwan, ROC; 5 Division of Nephrology, Department of Medicine, Taipei Veteran General Hospital, Taipei, Taiwan, ROC; 6 Department of Family Medicine, National Taiwan University Hospital, Taipei, Taiwan, ROC; 7 School of Public Health, National Taiwan University, Taipei, Taiwan, ROC; 8 Division of Cardiology, Department of Medicine, Taipei Medical University and Hospital, Taipei, Taiwan, ROC; 9 Institute of Biomedical Engineering, National Tsing-Hua University, Hsinchu, Taiwan, ROC; 10 Institute of Cellular and System Medicine, National Health Research Institute, Zhunan, Taiwan, ROC; 11 Present Address: Cardiovascular Center, National Taiwan University Hospital Hsinchu Branch, No. 25, Lane 442, Sec. 1, Jingguo Rd, Hsinchu City, 300 Taiwan

**Keywords:** Interventional cardiology, Haemodialysis

## Abstract

This study aimed to compare stent graft with balloon tamponade for ruptured dialysis access during percutaneous transluminal angioplasty. Patients over an 8-year period (2010–2018) were identified from a database of 11,609 procedures. The primary endpoint was target lesion primary patency at 12 months. A total of 143 patients who had rupture dialysis access were enrolled, of whom 52 were salvaged by stent grafts and 91 were salvaged by balloon tamponade. The 6-month target lesion primary patency was greater in the stent graft group than in the balloon tamponade group (66.7% vs. 29.5%, *P* < 0.001). The benefit of stent grafts was sustained for 12 months (52.5% vs. 9.0%, *P* < 0.001). The stent grafts increased the median time from the index procedure to the next intervention in the ruptured area by 171 days (260 vs. 89 days) at 12 months. There was no significant difference in the access circuit patency rates at 6 months (25.5% vs. 19.8%, *P* = 0.203) and 12 months (12.0% vs. 5.8%, *P* = 0.052). The patency results of the stent grafts remained after the multivariable adjustment analysis. Compared to balloon tamponade alone, stent grafts provided superior target lesion primary patency at 6 and 12 months. The access circuit patency rates were similar.

## Introduction

According to the 2019 National Kidney Foundation Kidney Dialysis Outcome Quality Initiative (KDOQI) clinical practice guidelines, percutaneous transluminal angioplasty (PTA) is considered as the primary treatment for vascular access dysfunction^1^. Although PTA is an established treatment, complications may develop during the procedure. Venous rupture is the most common complication during PTA, ranging from 1.7% to 14.9%^2–4^. Risks become higher with increasing lesion complexity and application of high-pressure balloon dilatation.

Various techniques have been used to treat PTA-related ruptures, including balloon tamponade (BT), stent placement, and intentional thrombosis. PTA-related venous rupture is usually salvaged by BT, followed by stent placement for uncontrolled bleeding. The reported success rate of these techniques varied from 62 to 100%^2^. Nonetheless, the patency of ruptured vessels is poor, ranging from 20 to 40% only, even when bare-metal stents are used^2,4–6^. This patency rate is well below the reasonable goal of patency at 6 months stated in the international guidelines for vascular access^1,7^. High restenosis rates implicated that BT may be a considerable therapy for immediate management, but not an effective one for maintaining the patency of the ruptured area.

Stent grafts (SGs) are the primary therapy for ruptured coronary or peripheral arteries. The use of SG to seal ruptured dialysis vascular access has been reported in sporadic cases^8,9^. In addition to serving as a vascular patch to seal the tear, the polytetrafluoroethylene (PTFE) graft also provides a mechanical barrier to prevent neointimal tissue ingrowth. In previous randomized controlled trials, SGs have prevented restenosis at the venous anastomosis of dialysis grafts^10,11^. The effect of SGs on the patency of ruptured dialysis access has not been comprehensively evaluated. In this study, we aimed to evaluate the immediate and long-term patency outcomes of SGs compared to BT in treating vessel ruptures induced by PTA of dialysis vascular accesses.

## Results

### Patient enrollment

**Figure 1:**
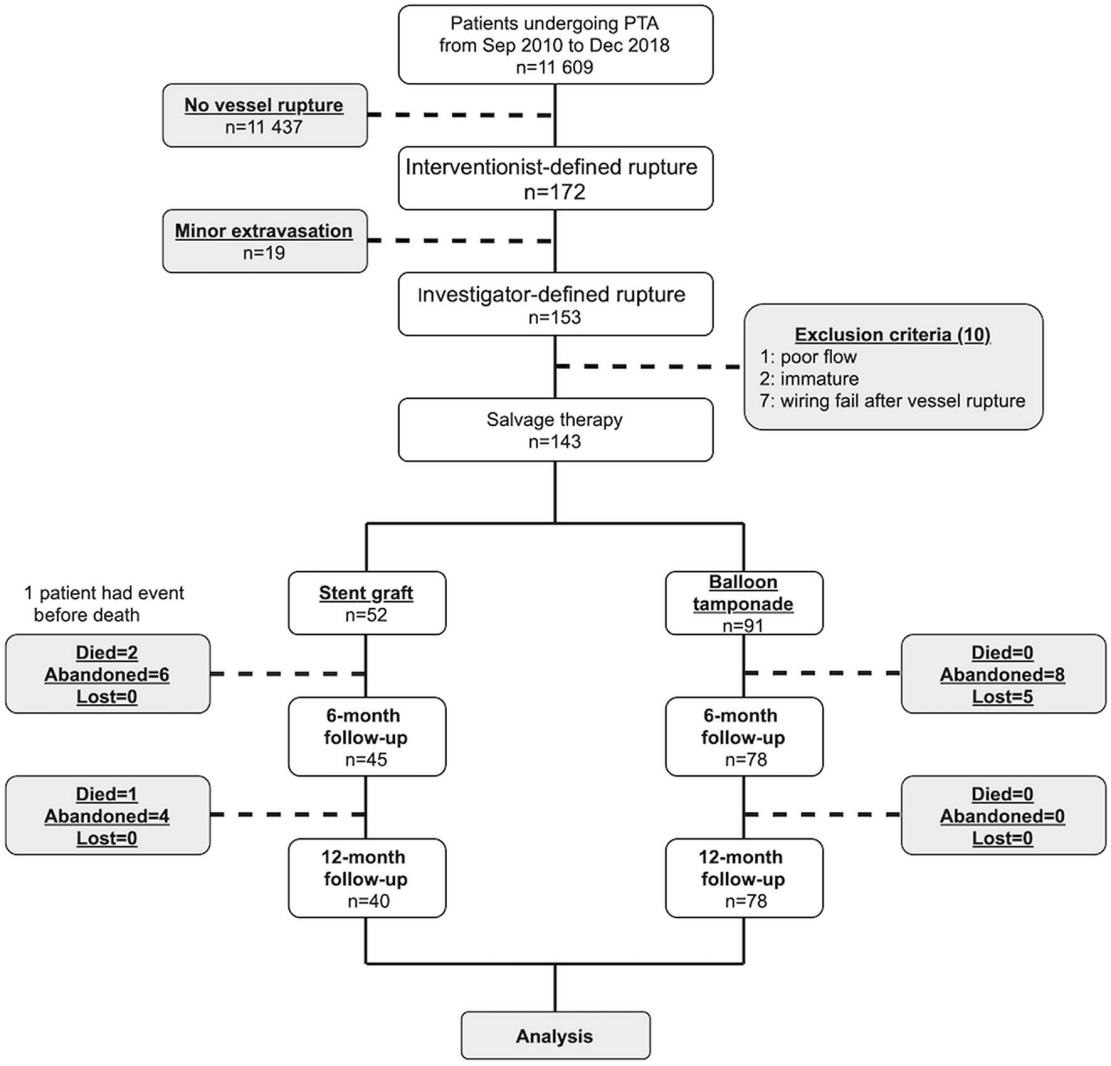
Flow diagram of the study subjects. *PTA* percutaneous transluminal angioplasty.

From September 2010 to December 2018, 172 procedures coded with rupture complications (interventionist-defined rupture) were identified from 11,609 PTA procedures in the computerized database. The angiograms were reviewed by one interventionist who had 18-year experience in endovascular therapy of dialysis vascular access. After reviewing the angiograms, 19 vessel ruptures with only a tiny area of contrast extravasation were excluded (investigator-defined rupture). After reviewing 153 procedure notes, the following 10 procedures were excluded: one due to poorly developed outflow veins, two due to immature access circuits, and 7 due to wiring failure. During the 12-month follow-up period, three patients died, five were lost to follow-up, and 18 had dialysis access abandonment. Two patients in each group underwent SG placement during reinterventions during the follow-up period. The final analysis consisted of 143 procedures involving vessel rupture (Fig. 1).

### Study participants

Among the 143 patients enrolled, 52 were salvaged by SG and 91 were salvaged by BT alone. The SG group had more procedures that encountered total occlusion (53.8% vs. 34.1%, *P* = 0.017) and multiple stenoses (46.2% vs. 24.2%, *P* = 0.009) than the BT group. The SG group had more basilic vein rupture (57.7% vs. 38.9%) and cephalic arch rupture (23.1% vs. 11.1%) than the BT group. The balloon size of the SG group was larger than that of the BT group (7.3 vs. 6.9 mm, *P* = 0.016). No differences were observed in other baseline characteristics. (Table 1). According to the description of the procedure notes, the decisions for SG placement were due to persistent bleeding for 17 vascular accesses and based on interventionists’ choice for 35 vascular accesses.

**Table 1:** Baseline characteristics of study participants according to treatment group.

Characteristics	SG	BT	*P* value
	n = 52	n = 91	
**Demographic data**			
Age (year)	75.8 ± 12.7	75.4 ± 11.7	0.844
Male (%)	15 (28.8%)	38 (41.8%)	0.086
Hypertension	38 (73.1%)	58 (63.7%)	0.169
Diabetes mellitus	21 (40.4%)	32 (35.2%)	0.328
CAD	27 (51.9%)	47 (51.6%)	0.557
Smoking	1 (1.9%)	8 (8.8%)	0.098
Dyslipidemia	12 (21.3%)	13 (14.3%)	0.136
**Access data**			
Shunt age (months)	25 (12–57)	18 (12–48)	0.404
Native/graft	21/31	41/50	0.358
Left/right arm	44/8	75/16	0.464
Forearm/upper arm	37/15	70/21	0.294
**Lesion data**			
Total occlusion	28 (53.8%)	31 (34.1%)	0.017
Multiple lesions	24 (46.2%)	22 (24.2%)	0.009
**Rupture site**			0.015
Radial artery	1 (1.9%)	1 (1.1%)	
Anastomosis complex	0	9 (10.0%)	
Outflow vein	46 (88.5%)	65 (71.4%)	
Graft or graft-vein junction	5 (9.6%)	15 (16.5%)	
**Rupture flow**			0.749
No flow	17 (32.7%)	25 (27.5%)	
Slow flow	13 (25.0%)	22 (24.2%)	
Normal flow	22 (42.3%)	44 (48.4%)	
**Procedural data**			
PTD	3 (5.8%)	2 (2.2%)	0.254
Cutting balloon	1 (1.9%)	0	0.364
Balloon diameter	7.3 ± 1.0	6.9 ± 0.8	0.016
Balloon length	51.2 ± 27.8	44.0 ± 22.9	0.116
Pre-stenosis (%)	76.3 ± 11.6	74.8 ± 12.5	0.564

Values are expressed as n (%), mean ± SD, or median (IQR).

*SG* stent graft, *BT* balloon tamponade, *CAD* coronary artery disease, *PTD* percutaneous thrombectomy device.

### Immediate outcomes

The procedure time in the SG group was longer than that in the BT group (median, 33 min vs. 23 min, *P* = 0.016). The post-intervention stenosis of the SG group was lower than that of the BT group (2.9 ± 7.2% vs. 17.8 ± 19.7%, *P* < 0.001). In the SG group, one patient experienced procedure failure, while the other had recurrent thrombosis before the next dialysis session. In the BT group, six patients experienced procedure failure (Table 2). All patients with clinical success received dialysis from their vascular access without the need for interim catheters. One patient in the SG group experienced vascular access infection 4 days after the procedure. There was no significant arm edema due to outflow vein loss or skin erosion due to SG. Except for the 18 abandoned vascular accesses and five patients who were lost to follow-up, all the other 120 vascular accesses were used for dialysis 1 year after the procedure.

**Table 2:** Comparison of immediate and patency outcomes.

Outcomes	SG	BT	*P* value
	n = 52	n = 91	
**Immediate outcome**			
Procedure time (min)	33 (23–60)	23 (17–42)	0.016
Post-stenosis (%)	2.9 ± 7.2	17.8 ± 19.7	< 0.001
Anatomical success	51/52 (98.1%)	85/91 (93.4%)	0.710
Clinical success	50/52 (96.2%)	85/91 (93.4%)	0.990
**Patency outcome**			
Target lesion primary patency rate (%)			
6 months	30/45 (66.7%)	23/78 (29.5%)	< 0.001
12 months	21/40 (52.5%)	7/78 (9.0%)	< 0.001
Access circuit primary patency rate (%)			
6 months	13/51 (25.5%)	17/86 (19.8%)	0.203
12 months	6/50 (12.0%)	5/86 (5.8%)	0.052
Access circuit assisted primary patency (%)			
6 months	35 (67.3%)	63 (69.2%)	0.477
12 months	33 (63.5%)	56 (61.5%)	0.482
Secondary patency rate (%)			
6 months	46 (88.5%)	83 (91.2%)	0.398
12 months	42 (80.8%)	83 (91.2%)	0.063
Number of reinterventions months			
6 months	1 (0–2)	1 (0–2)	0.242
12 months	3 (1–5)	2 (1–4)	0.570

Data are presented as n (%), median (IQR), or mean ± SD.

*SG* stent graft, *BT* balloon tamponade.

### Patency outcomes

**Figure 2:**
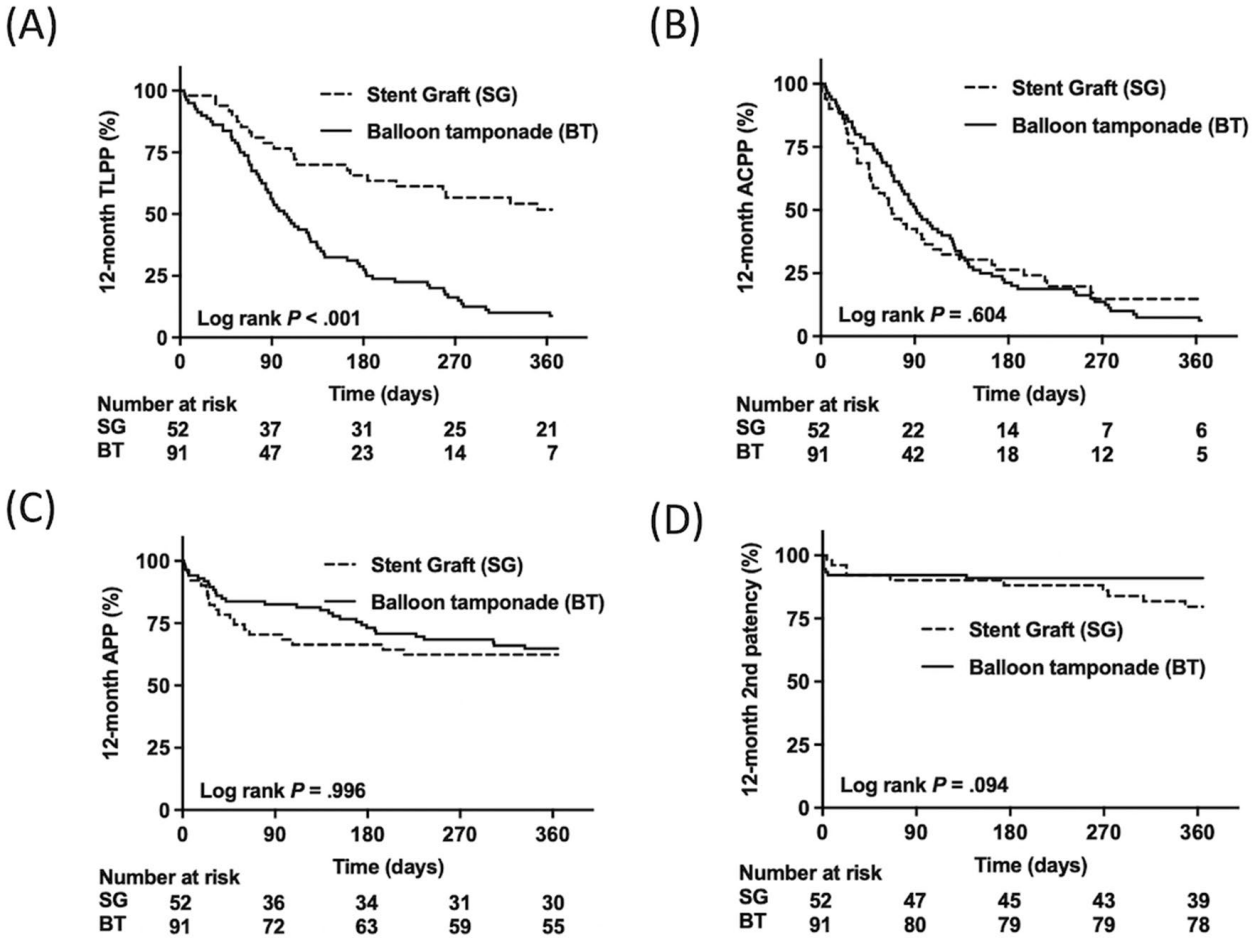
Kaplan–Meier plots of the cohort until 12 months: (**A**) target lesion primary patency, (**B**) entire access circuit primary patency, (**C**) access circuit assisted patency, and (**D**) secondary patency after the interventions. *SG* stent graft, *BT* balloon tamponade, *TLPP* target lesion primary patency, *ACPP* access circuit primary patency, *APP* access circuit assisted patency.

**Figure 3:**
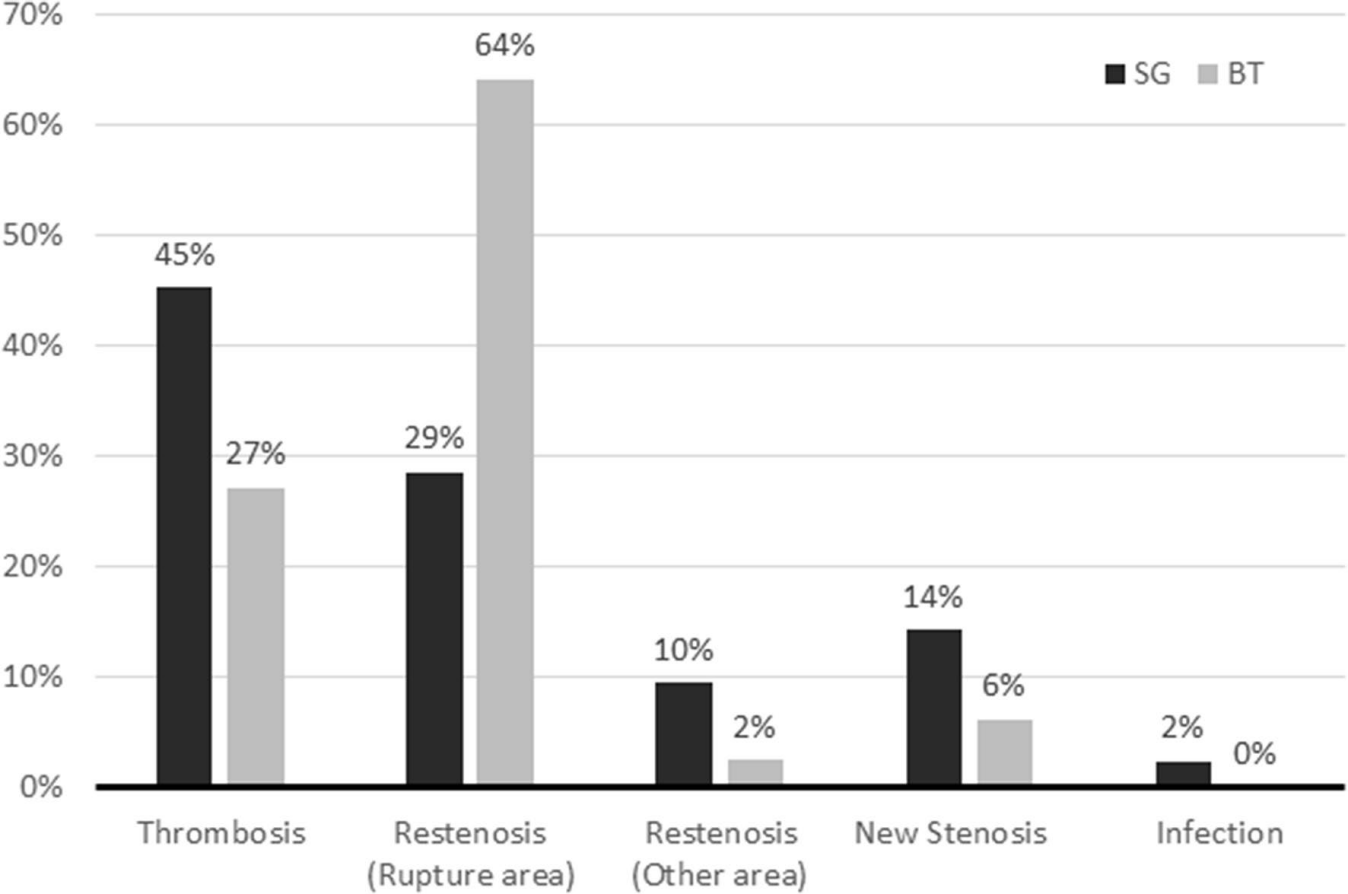
Proportions of patients with different causes of primary access circuit failure, stratified by the treatment modality for ruptured vessels. SG, stent graft; BT, balloon tamponade.

The patency outcomes of the SG and BT groups are shown in Table 2. The target lesion intervention-free intervals in the SG group were longer than those in the BT group at both 6 months (median, 180 vs. 89 days, *P* < 0.001) and at 12 months (260 vs. 89 days, *P* < 0.001). The access circuit intervention-free intervals were similar between groups at both 6 months (SG vs. BT, 65 vs. 80 days, *P* = 0.990) and 12 months (SG vs. BT, 65 vs. 80 days, *P* = 0.998). The primary patency rate of the target lesion in the SG group was higher than that in the BT group at 6 months (66.7% vs. 29.5%, *P* < 0.001) and 12 months (52.5% vs. 9.0%, *P* < 0.001). The access circuit primary patency rates were similar between groups at 6 months (SG vs. BT, 25.5% vs. 19.8%, *P* = 0.203) and 12 months (SG vs. BT, 12.0% vs. 5.8%, *P* = 0.052). The patency outcomes are also demonstrated by Kaplan–Meier plots (Fig. 2). The most common cause of access circuit primary patency loss was restenosis in the ruptured area in the BT group and thrombosis in the SG group. The SG group also had a higher proportion of restenosis in the non-ruptured area and de novo stenosis than the BT group (Fig. 3).

There was no significant difference in the access circuit-assisted primary patency rates between the groups at both 6 months (SG 67.3% vs. BT 69.2%, *P* = 0.477) and 12 months (SG 63.5% vs. BT 61.5%, *P* = 0.482). There was no significant difference in the secondary patency rates between the groups at both 6 months (SG 88.5% vs. BT 91.2%, *P* = 0.398) and 12 months (SG 80.8% vs. BT 91.2%, *P* = 0.063) after the procedure. There was no significant difference in the number of reinterventions between the groups within 12 months after the procedure.

### Univariable and multivariable analysis

Univariable Cox regression analysis showed that the HR of the primary patency of the target lesion in the SG group compared to the BT group was 0.322 at 6 months (95% confidence interval [CI], 0.186–0.558; *P* < 0.001) and 0.288 at 12 months (95% CI, 0.178–0.466; *P* < 0.001) (Table 3). Multivariable Cox regression analysis showed that the benefit of target lesion primary patency remained at both 6 months (HR, 0.290; 95% CI, 0.155–0.541; *P* < 0.001) or 12 months (HR, 0.308; 95% CI, 0.179–0.529; *P* < 0.001), after adjusting for age, sex, hypertension, diabetes mellitus, coronary artery disease, smoking, dyslipidemia, shunt age, native, left/right, forearm/upper, total occlusion, multiple lesions, rupture flow, percutaneous thrombectomy device, cutting balloon, balloon diameter, and balloon length (Table 3).

**Table 3:** Cox proportional hazard ratio of patency: patients with rupture dialysis access undergoing stent graft in comparison with balloon tamponade.

Outcomes	Crude		Fully adjusted*	
	HR (95% CI)	*P* value	HR (95% CI)	*P* value
**Primary analysis**				
Target lesion primary patency				
6 months	0.322 (0.186–0.558)	< 0.001	0.290 (0.155–0.541)	< 0.001
12 months	0.288 (0.178–0.466)	< 0.001	0.308 (0.179–0.529)	< 0.001
Access circuit primary patency				
6 months	0.964 (0.646–1.439)	0.859	1.044 (0.647–1.685)	0.860
12 months	0.906 (0.624–1.317)	0.605	1.071 (0.687–1.671)	0.761
Access circuit assisted patency				
6 months	1.111 (0.608–2.031)	0.731	0.972 (0.482–1.961)	0.937
12 months	0.998 (0.571–1.746)	0.996	0.815 (0.416–1.596)	0.551
Access circuit secondary patency				
6 months	1.260 (0.437–3.634)	0.668	1.430 (0.392–5.217)	0.588
12 months	2.166 (0.854–5.492)	0.103	2.564 (0.817–8.045)	0.107
**Sensitivity analysis 1: Excluding patients with initial failure**				
Target lesion	0.304 (0.187–0.494)	< 0.001	0.364 (0.204–0.650)	0.001
Access circuit	0.957 (0.653–1.401)	0.820	1.256 (0.789–2.001)	0.337
**Sensitivity analysis 2: Excluding patients stented due to persistent bleeding**				
Target lesion	0.310 (0.177–0.543)	< 0.001	0.340 (0.174–0.667)	0.002
Access circuit	0.939 (0.617–1.428)	0.768	1.047 (0.617–1.778)	0.864
**Sensitivity analysis 3: Including patients with minor extravasation**				
Target lesion	0.292 (0.183–0.466)	< 0.001	0.312 (0.177–0.550)	< 0.001
Access circuit	0.890 (0.622–1.273)	0.522	1.123 (0.715–1.763)	0.615

Primary analyses: 6-month and 12-month target lesion and access circuit primary patency, access circuit assisted patency, and access circuit secondary patency; Sensitivity analyses: 12-month target lesion and access circuit primary patency.

*CI* confidence interval, *HR* hazard ratio.

*Adjusted by age, sex, hypertension, diabetes mellitus, coronary artery disease, smoking, dyslipidemia, shunt age, native, left/right, forearm/upper, total occlusion, multiple lesions, rupture flow, percutaneous thrombectomy device, cutting balloon, balloon diameter, balloon length.

### Sensitivity analyses and subgroup analyses

All three sensitivity analyses showed similar results, either target lesion primary patency or access circuit primary patency (Table 3 and Supplement Fig. S1). The Cox regression analysis in the subgroup of patients showed a similar pattern of the target lesion and access circuit patency at both 6 months and 12 months (Supplement Fig. S2). There was no significant between-group difference in the target lesion and access circuit patency at both 6 months and 12 months according to age, sex, shunt type, total occlusion, multiple lesions, and rupture flow.

## Discussion

### Main findings

The primary patency of ruptured vessels observed in this study was initially 29.5% only at 6 months, well below the 50% goal set by the KDOQI guidelines^1^. Our analysis revealed that SGs could improve the patency of ruptured areas by up to 66.7%. The superiority in patency was sustained for 12 months in the ruptured area. In dialysis access interventions, evidence favoring SGs is available only for venous anastomosis of dialysis grafts^10,11^. Our study demonstrated that SGs provided patency benefits in the ruptured vessels of dialysis access.

Vessel rupture is the most common complication of dialysis access interventions, accounting for nearly 70% of the complications^12^. BT is traditionally used as first-line therapy, followed by bare-metal stent, if bleeding cannot be controlled^2^. Treatment success ranges from 68 to 100% (average of 82%)^2^. The advantage of an SG over the conventional method is a complete physical barrier to prevent extravasation from the torn vessel. Our data demonstrated that bleeding could be well-controlled in a relatively large number of patients by placing an SG to cover the ruptured vessel.

Nonetheless, the effectiveness of a technique or device depends not only on the immediate results, but also on the durability of its effect. Therefore, reasonable goals for patency after endovascular interventions are recommended by various societies^1,7,13^. Currently, the patency of ruptured vessels salvaged by PTA is far below the goals recommended by such guidelines. A review of studies by Trerotola et al. described a 6-month primary patency rate of 20–40% only, even when bare-metal stents were used^2^. Our study specifically evaluated the patency at the ruptured area (29.5%) and overall access circuit (19.8%) at 6 months using conventional methods, both of which were far below the recommended goal (50%). These data suggest that ruptured vessels are at an extremely high risk for reintervention.

An SG can resist elastic recoil, optimize luminal diameter, and provide a physical barrier to prevent ingrowth of neointimal tissues. Previous studies have demonstrated that SGs improved patency in certain circumstances of high restenosis risk, such as stenosis at the graft-venous anastomosis and cephalic arch^10,11,14^. Currently, only one case series described access circuit patency outcomes of SG therapy for vessel ruptures^8^. Nonetheless, the outcome at the ruptured area has not been specifically evaluated. Our study evaluated both the ruptured area and the entire access circuit. To evaluate a specific device, the concerning area should be the primary focus of interest. Our study is the first to evaluate the effects of SG on ruptured vessels. We found a significant improvement in patency by 91 days at 6 months and prevented reinterventions by 68%. The benefit of patency was sustained for 12 months. The effect size on patency was also similar to the effect of SG on venous anastomosis stenosis of dialysis grafts^10,11^. Our results also implied that the benefit of SGs may be extended to native vessels at a high risk for restenosis.

Despite the non-randomized nature of our retrospective study, the superiority of SG may be underestimated by a variety of unfavorable selection biases. For example, SGs are usually reserved for difficult-to-control bleeding cases or cases that experience unfavorable results after BT. The SG group in this study also had more risk factors for restenosis than the BT group, such as total occlusion, multiple stenoses, and no flow after rupture. However, the patency of the SG group was still significantly superior to that of the BT group. Even after meticulous adjustment of confounding factors using multivariable analysis, the benefits of SG remained. The SG group had a higher immediate procedure success than the BT group, which may have biased the evaluation of patency outcomes. After excluding cases of initial procedure failure, the benefit of SG on patency alone remained in the sensitivity analysis.

Given the baseline characteristics of our cohort, it was not unexpected that the patency of the entire access circuit did not improve with SG placement, either primary or secondary patency. As demonstrated in Table 1, a higher percentage of the SG group experienced conditions such as total occlusion and multiple lesions, as compared to the BT group. Of the SG group, 54% had total occlusion (34% in the BT group), a well-known risk factor for patency loss; 46% had multiple stenoses (24% in the BT group). In the analysis of access circuit primary failure, thrombosis, non-target lesion restenosis, and de novo stenosis accounted for 69% of the causes for receiving reinterventions, suggesting a poor underlying vascular condition in the SG cohort. As demonstrated in Fig. 3, the proportion of subsequent access thrombosis of the SG group is higher than that of the BT group. The discrepancy in subsequent thrombosis is likely due to difference in baseline vascular conditions or thrombogenic risk of stent graft^15,16^. We tried to identify whether a certain subgroup would benefit from SG placement for access circuit outcomes, but none of these subgroups showed a beneficial effect. If this finding persisted with proper randomization, any possible benefit of SGs for access circuit patency would be eliminated. In future studies, a more comprehensive evaluation and treatment of various pathogenic pathways may be needed to achieve better outcomes for the entire access circuit. There was no significant difference in the secondary patency, which depends on multiple factors rather than the target lesion alone.

### Limitation

This study had some limitations. First, this was a retrospective study, in which patients were included with a selection bias. Second, misclassification bias should also be considered, as some patients in the BT group were not suitable for SG because of rupture locations. Third, information bias could not be avoided because of the retrospective nature of the data collection. For example, the identified ruptures of 172 out of total of 11,609 procedures is only 1.48% rate of ruptures. This very low rate of ruptures suggests that some cases might not be coded in the database. Fourth, drug-coated balloons improved post-interventional patency in recent meta-analyses and clinical trials^17–19^. The difference between SG and BT groups may be different if drug-coated balloons are used. Finally, it is not possible to make a general recommendation for SG use because of conflicting results in target lesion patency and access circuit patency. Nonetheless, a balanced presentation of scientific evidence and limitations may help justify SG use in different situations.

## Conclusion

Our study demonstrated that SG placement effectively prolonged the target lesion patency of ruptured vessels from 29.5% to 66.7% at 6 months. The patency benefit on the ruptured area was sustained for 12 months after the intervention. Nonetheless, no significant benefit on the overall access circuit patency was found.

## Methods

### Study design and enrollment of patients

This single-center, retrospective study was approved and informed consent was waived by the Institutional Review Board of the National Taiwan University Hospital, Hsinchu Branch. All methods were carried out in accordance with relevant guidelines and regulations. All the information of the formatted PTA procedure notes, including clinical (age, sex, dialysis duration, comorbidities, and medication), access (age, type, location, side, and indication for PTA), lesion data (location, severity, and length), and interventional data (stenosis before and after therapy, success, procedure time, device, complication, and management), were converted into digital data at monthly intervals, which were stored in a computerized database and maintained by the institutional director of the endovascular intervention team.

Eligible cases were identified from the PTA database using the complication item, “rupture,” (interventionist-defined rupture) and stratified by the device item, “stent graft,” spanning from 9/1/2010 to 12/31/2018. After identifying the target patients, the location of rupture and blood flow after rupture were evaluated by conducting a retrospective review of angiography. Rupture was defined as extravasation of contrast medium at the PTA site requiring treatment; a tiny area of extravascular contrast medium that was not enlarged was considered an expected outcome of PTA and was not included (investigator-defined rupture). The outcomes of patients, access circuits, and target lesions were evaluated by conducting a retrospective review of dialysis records, angiograms, and procedure notes within 1 year after the intervention. Procedures were excluded from the final analysis due to one of the following: (1) immature access for less than 3 months, (2) wiring failure, and (3) poorly developed outflow veins. (Fig. 1).

### Devices

Both semi-compliant (Wanda and Mustang, Boston Scientific, Galway, Ireland; Fox and Armada, Abbott, Diegem, Belgium) and non-compliant balloons (Conquest, Bard, Crawley, UK) were available in our angiographic unit. The choice of PTA balloon depended on the discretion of the physician. Since 2013, SGs have been approved by the Food and Drug Administration to treat venous anastomosis of arteriovenous grafts. Additionally, treatment costs can be reimbursed by the national health insurance system of Taiwan for vessel rupture or graft venous restenosis. The Viabahn SG (W. L. Gore & Associates, Flagstaff, Ariz) consists of a self-expanding, PTFE stent. Three different lengths of this stent (50, 100, and 150 mm), with diameters ranging from to 6–9 mm, were used at our institution.

### Procedures

Standard endovascular interventions were performed according to a previously published study^20^. The lesion was crossed with a 0.035-inch, hydrophilic guide wire (Terumo, Tokyo, Japan) with an adequate PTA balloon (the same size or 1 mm larger than the diameter of the reference vessel). The initial balloon was inflated at the nominal pressure. If a waist in the balloon persisted, the pressure would be escalated until the waist was effaced or the rated burst pressure was reached. If the waist could not be effaced or residual stenosis was > 30%, a non-compliant balloon, or a cutting balloon (Peripheral Cutting Balloon, Boston Scientific, Natick, MA), was used to efface the lesion. Drug-coated balloons were not used in this study. For thrombosed vascular access, endovascular thrombectomy was modified from the techniques reported by Trerotola et al.^21^, who used mechanical thrombectomy devices (Arrow-Trerotola percutaneous thrombectomy device; Arrow, Reading, Pennsylvania) for long-segment or wall-adherent thrombi. No additional medical lytic therapy was used^15^. SGs were used only for vessel rupture or restenosis at the graft-venous junction during the study period, based on the regulations of the Health Insurance Bureau. After confirmation of antegrade flow, diagnostic fistulography was performed. Any stenoses in the outflow vein were identified and treated with PTA. The puncture site was manually compressed until hemostasis was achieved.

The rupture was initially managed by BT at our institution. Prolonged balloon inflation using a PTA balloon of the same size, at low pressure of 2 atm, 3 min per cycle, was repeated until proper management of contrast extravasation. If heparin was used, protamine was used to reverse the anticoagulant effect. Manual compression at the rupture site was used in some cases, but was not routinely recorded in the database. For ruptures not sealed by BT, intentional thrombosis was performed by external compression or balloon occlusion. When SG was available and may be reimbursed, the decision to use BT alone or SG placement depended on the physician. SG deployment was avoided in the puncture zone of vascular access. The size of the covered stents was similar to that of PTA balloons. The system was advanced over a 0.035-inch guidewire (Terumo, Tokyo, Japan) via a 7-Fr or 8-Fr vascular sheath (Terumo, Tokyo, Japan), depending on the stent size. During the exchange for a larger sheath or stiff wire before the stent was ready, manual compression of the rupture site may be needed, as assisted by the angiographic unit personnel.

### Follow-up

After the intervention, patients were followed up in their respective hemodialysis centers based on a common surveillance protocol suggested by our nephrology society, including physical examination, blood flow, and venous pressure monitoring in each dialysis session, as well as blood flow by ultrasound dilution method, if available. When abnormal hemodynamic parameters or clinical evidence meet the criteria for dysfunction (Supplemental Table S1), patients are referred for further evaluation. Intervention was indicated if a stenosis more than 50% with correlated clinical or hemodynamic abnormalities^15,20^. Follow-up data were obtained by our vascular access coordinator through telephone contact with referral centers at 3-month intervals. For this study, all PTA procedure notes and angiograms within 1 year after the index procedure were reviewed to confirm the outcome of the ruptured vessels. Target lesion restenosis was defined as > 50% diameter stenosis in the previously ruptured area.

### Definition of endpoints

Anatomical success was defined as < 30% residual stenosis of the treated vascular segment. For thrombosed accesses, anatomical success was defined as the restoration of flow, combined with < 30% residual stenosis. Clinical success was defined as the resumption of normal hemodialysis for a minimum of at least one session following the intervention. Post-interventional target lesion primary patency was defined as the time to the next intervention of the ruptured area. Post-interventional access circuit primary patency was defined as the time to thrombosis or the next intervention within the vascular access. Post-interventional access circuit-assisted primary patency was defined as the time to thrombosis or surgical intervention of the access circuit. Post-interventional secondary patency was defined as the time to surgical thrombectomy, revision, or abandonment of vascular access. All endpoints were defined based on the reporting standards of the Society for Vascular Surgery evaluating PTA in arteriovenous hemodialysis accesses^22^.

### Statistical analysis

For population characteristics, mean ± standard deviation measurements were used to describe continuous variables, which were compared using Student’s t-test and analysis of variance. The Mann–Whitney U test for abnormally distributed data was used to compare continuous variables. Numbers and percentages were used to describe the categorical variables, which were compared using the chi-square test if numbers were greater than 5 and the Fisher’s exact test if numbers were below 5. Multivariable Cox proportional hazard ratio (HR) analysis was performed for the original cohort, adjusting for age, sex, hypertension, diabetes, coronary artery disease, smoking, dyslipidemia, shunt age, access type, access location, multiple lesions, occlusion, rupture flow, and devices used. Kaplan–Meier survival analysis was used to estimate the proportional outcomes of the target lesion and entire access primary patency at the 6-month and 12-month follow-ups. Subgroup analysis with age, sex, arteriovenous graft (AVG) or arteriovenous fistula (AVF), total occlusion, multiple lesions, and rupture flow was performed. Three sensitivity analyses were performed: (1) excluding patients with failed initial procedure in order to observe the patency effect alone; (2) excluding patients who had received stent placement due to persistent bleeding in order to remove situations without options for SG choice; and (3) including patients with only a tiny area of extravascular contrast medium on angiogram (interventionist-defined rupture) in order to eliminate selection bias. Statistical analyses were performed using SPSS version 22.0 for Windows (SPSS Inc., Chicago, IL, USA) and R Statistics version 3.6.2 for Windows (The R Foundation for Statistical Computing, Vienna, Austria).

## Supplementary Information

Supplementary Figures.

Supplementary Table 1.
