# Supplementary material for: Stent grafts improved patency of ruptured hemodialysis vascular accesses
Source: Sci Rep. 2022 Jan 7;12:51. doi: 10.1038/s41598-021-03933-1 (PMC8741950; doi:10.1038/s41598-021-03933-1)
Supplement: Supplementary file 1 — Supplementary Figures. [file 41598_2021_3933_MOESM1_ESM.docx]

Supplement Figure 1 Kaplan-Meier plots of 12-month primary patency by three sensitivity analyses: Target lesion (A) and access circuit (B) primary patency of the cohort excluding patients with initial procedure failure in sensitivity analysis 1; target lesion (C) and access circuit (D) primary patency of the cohort excluding patients stented due to persistent bleeding in sensitivity analysis 2; target lesion (E) and access circuit (F) primary patency of the cohort including patients with minor extravasation (interventionist-defined ruptures) in sensitivity analysis 3. *SG* stent graft, *BT* balloon tamponade, *TLPP* target lesion primary patency, *ACPP* access circuit primary patency.





Supplement Figure 2 Forest plots of subgroup analysis: (A) 6-month and (B) 12-month target lesion primary patency and (C) 6-month and (D) 12-month entire access circuit primary patency after the interventions. *SG* stent graft, *BT* balloon tamponade, *AVG* arteriovenous graft, *AVF* arteriovenous fistula.
