# Supplementary material for: Stent grafts improved patency of ruptured hemodialysis vascular accesses
Source: Sci Rep. 2022 Jan 7;12:51. doi: 10.1038/s41598-021-03933-1 (PMC8741950; doi:10.1038/s41598-021-03933-1)
Supplement: Supplementary file 2 — Supplementary Table 1. [file 41598_2021_3933_MOESM2_ESM.docx]

**Supplemental Table 1.** Surveillance protocol, referral criteria, and intervention criteria for hemodialysis vascular access in this cohort

| **Surveillance protocol** |  |
| --- | --- |
|  | Physical examination (each session) |
|  | Dynamic venous pressure monitoring (each session) |
|  | Dialysis dose (monthly) |
|  | Access blood flow (monthly, if available) |
| **Referral criteria** |  |
|  | Physical examination |
|  | - Diminished or abnormal thrill |
|  | - Pulsatility |
|  | - Flaccid access |
|  | - Abnormal bruits |
|  | - Arm or hand swelling |
|  | Clinical manifestation |
|  | - Prolonged bleeding |
|  | - Difficult puncture |
|  | - Pulling clots |
|  | Blood flow by ultrasound dilution method |
|  | - Total blood flow <500 ml/min for arteriovenous fistula, <600 ml/min for arteriovenous graft |
|  | - Reduction >25% from baseline |
|  | Dynamic venous pressure |
|  | - exceeding threshold level three consecutive times |
|  | Dialysis dose |
|  | - unexplained decrease >0.2 unit |
| **Intervention criteria** | 1. **and (B)** |
|  | 1. A lesion > 50% diameter stenosis |
|  | 1. Presence of at least one of the above referring criteria attributable to the stenosis |
